# Housing Insecurity and the Emotional and Physical Health of African Americans

**DOI:** 10.1093/geroni/igaa057.1076

**Published:** 2020-12-16

**Authors:** Aarti Bhat, August Jenkins, David Almeida

**Affiliations:** 1 Pennsylvania State University, Pennsylvania State University, Pennsylvania, United States; 2 Penn State, University Park, Pennsylvania, United States; 3 Pennsylvania State University, University Park, Pennsylvania, United States

## Abstract

Housing insecurity—or limited and/or unreliable access to quality housing— is a potent on-going stressor that can adversely impact individual well-being. This study extends previous research by investigating the impact of housing insecurity on both the emotional and physical health of aging African American adults using the Midlife in the United States (MIDUS) Refresher oversample of African Americans collected from 2012-2013 (N = 508; M age = 43.02; 57% women). Participants reported on their negative affect, number of chronic health conditions experienced in the last year, and experiences of housing insecurity since the 2008 recession (e.g., homelessness, threatened with foreclosure or eviction, lost home). Negative affect and chronic conditions, respectively, were regressed on housing insecurity, and the potential moderating effect of age was tested. Results showed that housing insecurity was associated with more negative affect (B = 0.05, SE = 0.03, p = .002) and chronic health conditions (B = 0.26, SE = 0.03, p < .001). Additionally, the association between housing insecurity and negative affect was moderated by age (B = -0.11, SE = 0.00, p = .019), such that the effect of housing insecurity on negative affect was stronger for younger adults than for older adults. These results suggest that experiences of insecure housing leave African American adults vulnerable to compromised emotional and physical health, however, the negative effects of housing insecurity may attenuate with age.

